# Patient-reported education regarding hand hygiene and use of non-sterile clinical gloves in an emergency department observation unit

**DOI:** 10.1016/j.infpip.2025.100501

**Published:** 2025-11-24

**Authors:** Hanna-Leena Melender, Elina Koota, Katariina Kainulainen, Karoliina Aho, Marja Mäkinen, Johanna Kaartinen

**Affiliations:** a Shared Group Services, Helsinki University Hospital, Helsinki, Finland; b University of Helsinki, Helsinki, Finland; c Research Unit of Health Sciences and Technology, University of Oulu, Oulu, Finland; d Emergency Medicine and Services, Helsinki University Hospital, Helsinki, Finland; e Division of Infectious Diseases, Inflammation Centre, Helsinki University Hospital, Helsinki, Finland

**Keywords:** Cross-sectional study, Emergency department observation unit, Hand hygiene, Non-sterile clinical gloves, Patient education, Standard precaution

## Abstract

**Background:**

Patient education regarding hand hygiene (HH) and the correct use of non-sterile clinical gloves (NSCGs) are important parts of infection prevention and control. Unnecessary use of NSCGs can be harmful, has associated financial costs, and harms the environment. This study aimed to explore healthcare workers' (HCWs) adherence to patient education regarding HH and the correct use of NSCGs in an observation unit.

**Methods:**

Data in this observational descriptive cross-sectional study were collected from patients using a questionnaire. The questionnaire asked about the patient education received and the use of NSCGs by HCWs. The correctness of NSCG use was determined by the investigators based on standard precautions on infection prevention and control. Statistical analysis and qualitative content analysis were performed.

**Results:**

The convenience sample consisted of 174 patients in an observation unit at Helsinki University Hospital, and 600 care, examination or test procedures conducted for patients. The response rate was 87%. Of the participating patients, 8.6% reported that they had received patient education on HH. Eighteen different procedures were conducted for the study patients. The use of NSCGs was always correct for six procedures. Unnecessary use of NSCGs was found (to varying degrees) for nine procedures, and insufficient use of NSCGs was found for three procedures. An association was found between a procedure/procedure type conducted for a patient and the correct use of NSCGs (*P*<0.001).

**Conclusions:**

Deviations from the standard precautions existed. Interventions for HCWs are needed to support routine patient education on HH and evidence-based use of NSCGs.

## Introduction

As part of a larger research project on the post-pandemic improvement of infection safety and reduction of adverse environmental effects, an observational descriptive cross-sectional study was conducted in an emergency department observation unit (henceforth ‘observation unit’) at Helsinki University Hospital. To support the aims of the project, this study investigated patient education on hand hygiene (HH) and the use of non-sterile clinical gloves (NSCGs) by healthcare workers (HCWs). Patients in the observation unit participated in this research.

### Patient education on HH

Patient education on HH is an important part of infection prevention and control. The World Health Organization [1] states that, in addition to the education of HCWs, the education of patients and visitors is critical for an enhanced safety climate in healthcare settings.

Research evidence on the effects of patient education regarding HH is promising. Rai *et al.* [2] found that an educational intervention encouraging patients to perform HH at five specific moments resulted in an increase in patient use of hand sanitizer that persisted for the following 3 days. Moreover, the intervention group performed HH when HCWs entered a room more often compared with the control group. In another study [3], an educational patient HH intervention resulted in reduced contamination of the hands of hospitalized patients with healthcare-associated pathogens. Desirable results were also gained in a study by Prokrywka *et al.* [4] who educated HCWs on how to teach patients about HH and provide them with opportunities to practice it. After the HCW education, both patient education regarding HH and opportunities offered for HH increased. *Clostridium difficile* infection events decreased during the study period.

Regardless of the good results of studies, a systematic review by Hammoud *et al.* [5] revealed a low percentage of patient education on infection control measures. The authors concluded that hospitals should emphasize the importance of patient engagement and education regarding infection control, and encourage patients to involve themselves in their care process by asking HCWs to provide them with information. In the observation unit of the study hospital, there was no clear picture of the state of patient education on HH; as such, one focus of this study was to discover whether any development efforts were needed.

### Use of NSCGs by HCWs

Although NSCGs are an important part of standard precautions regarding infection prevention and control, they are often not used correctly. For example, the overuse of NSCGs does not reduce the spread of multi-drug-resistant micro-organisms and may even increase the risk of infection [6,7]. Even before the coronavirus disease 2019 pandemic, studies had reported the unnecessary use of NSCGs [6,8,9]. One study revealed that, in 8.33% of observations, HCWs wore gloves even when it was not recommended to use them in that situation [10]. The additional financial costs caused by the overuse of NSCGs are remarkable.

Misuse of NSCGs affects infection prevention and control as well as having financial costs. It has been estimated that the healthcare share of carbon dioxide emissions is 6–7% [11]. Disposable gloves are one of the single-most used accessories in a hospital with the greatest burden on the environment [12].

HCW education and increased awareness regarding the proper use of personal protective equipment is an effective way to reduce the overuse of disposable products [13]. In addition, monitoring the frequency of use could be a novel way to motivate learning [14].

The social environment also appears to have an effect on HH among HCWs. Monsalve *et al.* [15] observed in-room and out-of-room HH opportunities for HCWs, and found that the observed adherence of a single HCW to HH increased when other HCWs were present. They also found that adherence increased with the number of HCWs nearby, but at a decreasing rate. Their results were consistent after controlling for possible confounding factors, different measures of social context, and different times of day.

To support the project, data were needed on the use of NSCGs by HCWs in order to determine whether any development efforts were needed. Thus, this became another focus of the study.

### Patient engagement in research

It has previously been agreed that both patients and the public should be involved in research whenever possible. They can add value by being involved, for example, in data collection for health service research by bringing their unique perspectives to the research [16]. Patient participation is ethical as it ‘democratizes’ the research process [17]. In a systematic review, Domecq *et al.* [17] found that engaging patients in research – at a preparatory, execution or translation phase – was feasible in most cases. They concluded that engagement may improve the credibility of the findings and their applicability to the target population, and may have an empowering effect on the participants. Moreover, they stated that any potential risks for engaging patients should be balanced against a wide range of potential benefits.

In the present study, patients in the observation unit were engaged in data collection by being invited to voluntarily observe the use of NSCGs by HCWs, and report their observations in a survey. Typically, infection control link nurses conduct HH observations in their own units. However, HCWs may sometimes adhere more strictly to HH practices when an infection control link nurse is nearby. To observe HH in a different way and to engage patients in research activities, they were invited to observe the use of NSCGs by HCWs. As another research interest concerned patient education on HH from the patient perspective, patients were asked about their experiences in the same survey. Thus, while the two topics of this study were not directly linked, both supported the aims of the larger project.

## Aim

This study aimed to explore HCW adherence to patient education regarding HH, and the correct use of NSCGs in an observation unit. The research questions were:

1. To what extent do patients receive patient education on HH in an observation unit?
2. To what extent is the use of NSCGs by the HCWs of the observation unit in accordance with standard precautions on infection prevention and control?
3. Is the correct use of NSCGs by the HCWs of the observation unit associated with the care, examination or test procedure conducted for the patient?
4. Is the correct use of NSCGs by the HCWs of the observation unit associated with the number of HCWs attending a care procedure, an examination or a test procedure conducted for the patient?

No hypotheses were prespecified. The ultimate goals were to produce up-to-date knowledge for use in the development of patient education on HH and in the de-implementation process of unnecessary NSCGs, and to test the method of collecting data based on reports by the patients in the observation unit.

## Methods

### Setting

The observation unit at Helsinki University Hospital treats over 9000 patients requiring 24–48 h observation for different acute complaints and diagnoses annually. As in other units, HCWs in the observation unit have been instructed to use standard precautions on infection prevention and control, which are based on instructions from the World Health Organization [1]. Over the data collection period, 1177 patients were treated in the observation unit.

### Data collection and sampling

Data for this observational descriptive cross-sectional study were compiled in September and October 2024, with a questionnaire developed for the purposes of the study. The research panel of the hospital, consisting of voluntary patients and clients, read through both the information sheet and the questionnaire and commented on their relevance and clarity. Both documents were amended based on the feedback received from the research panel.

The convenience sample consisted of 174 Finnish-speaking patients in one observation unit at Helsinki University Hospital. The study participants were recruited on weekdays (from Mondays to Fridays) by a research assistant, with the help of an assistant head nurse who indicated which patients were suitable for the study after careful consideration of the condition of each patient. The research assistant informed the patients about the study, and written consent was obtained from all participants. Patients on contact precautions were excluded. Of the recruited patients, many expressed positive feelings about the study and the opportunity to participate.

Two hundred patients who were eligible for study inclusion were contacted over 44 days; of these, 174 agreed to participate. Thus, the response rate was 87%. Patients who refused to participate reported that they were too tired or on strong pain medication and therefore not willing to participate.

The HCWs working in the observation unit were aware of the study. However, only the head nurses and the assistant head nurses were aware of the topic of the study.

### Questionnaire

Patients were asked if they had received education regarding HH from HCWs. If they answered ‘yes’, they had the opportunity to describe the content of the patient education in detail.

Patients were then asked to keep track of the use of NSCGs by HCWs over 1 day (24 h), up to five times, when the HCWs were providing care, an examination or a test procedure for the patient. Each procedure (1–5) was reported within its own section in the questionnaire. In each section, the patient was asked how many HCWs attended the procedure in question, and how many of them used NSCGs. Finally, patients were asked to describe, in their own words, the type of care, examination or test procedure being performed.

Background information on the demographic, clinical or social characteristics of the participants was not considered relevant to the study objectives.

### Data analysis

To describe the extent to which the patients had received education on HH, frequencies and percentages of ‘yes’ and ‘no’ answers were calculated. Descriptions on the content of the received patient education were analysed using inductive content analysis. Meaningful expressions of the answers were coded, and the codes were grouped based on the similarities of their content to categories which were named based on their content [18]. When reporting the content of received patient education, frequencies refer to how many times each type of content was mentioned in expansive responses.

To describe the extent to which the use of NSCGs by HCWs was in accordance with standard precautions, an inductive content analysis for descriptions of the care, examination or test procedure in question was first conducted, with the method described above [18]. Procedure categories were coded with numbers to allow for statistical analysis, and were saved in SPSS Statistics Version 29.0 (IBM Corp., Armonk, NY, USA). Next, the named procedure categories were categorized according to the standard precautions for infection prevention and control statement into one of two categories: 1=NSCGs must be used; or 0=NSCGs do not need to be used. Frequencies and percentages of the use and disuse of NSCGs for each procedure were calculated to show accordance with the standard precautions (Table I). In 48 (8.0%) cases, the patient had not indicated the procedure or the answer was unclear. These answers represent missing data.

**Table I:** The use of non-sterile clinical gloves (NSCGs) during procedures conducted for patients (*N*=600^a^)

Procedure/procedure type (care, examination or test)	Need to use NSCGs during the procedure	Number of procedures conducted correctly and incorrectly according to standard precautions
Taking a blood sample, frequency=118 (21.4%)	No	Used: 77 (65.3%) (incorrect)
		Not used: 41 (34.7%) **(correct)**
Clinical examination (with no contact with secretions, wounds or mucous membranes), frequency=102 (18.5%)	No	Used: 45 (42.1%) (incorrect)
		Not used: 57 (55.9%) **(correct)**
Administration of medication, fluids or blood products, frequency=98 (17.8%)	No	Used: 43 (43.9%) (incorrect)
		Not used: 55 (56.1%) **(correct)**
Clinical measurement, frequency=71 (12.9%)	No	Used: 18 (25.4%) (incorrect)
		Not used: 53 (74.6%) **(correct)**
Inserting, caring for and removing a cannula, frequency=61 (11.1%)	Yes	Used: 44 (72.1%) **(correct)**
		Not used: 17 (27.9%) (incorrect)
Helping with elimination of body wastes (in toilet or with a bedpan, urinary catheterization or removal of a urinary catheter, clearance of an ascites drainage bag) and secretion sampling, frequency=31 (5.6%)	Yes	Used: 27 (87.1%) **(correct)**
		Not used: 4 (12.9%) (incorrect)
Discussion with the patient (physicians, nurses), frequency=18 (3.3%)	No	Used: 6 (35.3%) (incorrect)
		Not used: 11 (64.7%) **(correct)**
Serving a meal, frequency=16 (2.9%)	No	Used: 13 (81.3%) (incorrect)
		Not used: 3 (18.8%) **(correct)**
Medical procedures demanding the use of sterile gloves (magnetic ureteric stent removal after pancreas transplant, taking a biopsy, insertion of a drain, punction), frequency=8 (1.4%)^b^	Yes	Used: 8 (100%) **(correct)**
		Not used: 0 (0%) (incorrect)
Transferring the patient/helping with moving or with a moving aid, frequency=7 (1.3%)	No	Used: 6 (85.7%) (incorrect)
		Not used: 1 (14.3%) **(correct)**
Examination of mucous membrane area (rectum, urinary tract, mouth), frequency=4 (0.7%)	Yes	Used: 4 (100%) **(correct)**
		Not used: 0 (0%) (incorrect)
Washing the patient (including intimate hygiene), frequency=4 (0.7%)	Yes	Used: 4 (100%) **(correct)**
		Not used: 0 (0%) (incorrect)
Wound care, frequency=4 (0.7%)	Yes	Used: 4 (100%) **(correct)**
		Not used: 0 (0%) (incorrect)
Inspection or maintenance of telemetry, frequency=4 (0.7%)	No	Used: 1 (25%) (incorrect)
		Not used: 3 (75%) **(correct)**
Making the bed, frequency=3 (0.5%)	Yes	Used: 1 (33.3%) **(correct)**
		Not used: 2 (66.7%) (incorrect)
Cleaning the floor, frequency=1 (0.2%)	Yes	Used: 1 (100%) **(correct)**
		Not used: 0 (0%) (incorrect)
Putting a nasal oxygen cannula on the patient, frequency=1 (0.2%)	No	Used: 1 (100%) (incorrect)
		Not used: 0 (0%) **(correct)**
Protection of skin or an organ (not intimate), frequency=1 (0.2%)	No	Used: 0 (0%) (incorrect)
		Not used: 1 (100%) **(correct)**

a Missing data: frequency=48 (8.0%).

b The patients could not be expected to differentiate between the use of sterile and non-sterile gloves; thus, it was expected that sterile gloves were used during the procedure.

To explore if the correct use of NSCGs was associated with the care, examination or test procedure conducted for the patient, comparisons of the variables between groups were conducted using Chi-squared test. The significance level was set at 5% (*P*<0.05). Procedure categories that were mentioned <15 times were excluded from the analysis.

To explore if the correct use of NSCGs was associated with the number of HCWs attending a care, examination or test procedure conducted for the patient, a new variable was created with two categories: 1=procedure was conducted alone; or 0=procedure was conducted in the presence of other HCWs. This variable was cross-tabulated with the variable representing correctness of the use of NSCGs (Table II). Chi-squared test was used to compare the variables between groups. Logistic regression analysis was conducted to discover whether the number of HCWs attending a care, examination or test procedure explained the correct use of NSCGs.

**Table II:** Unnecessary and insufficient use of non-sterile clinical gloves (NSCGs) during the procedures conducted for patients (procedures mentioned <15 times were excluded from the compilation)

Unnecessary use of NSCGs (NSCGs were used when not indicated)	Insufficient use of NSCGs (NSCGs were not used when indicated)
Serving a meal (81.3%)	Inserting, caring for and removing a cannula (27.9%)
Taking a blood sample (65.3%)	Helping with elimination of body wastes and secretion sampling (12.9%)
Administration of medication, fluids or blood products (43.9%)	-
Clinical examination (42.1%)	-
Discussions (35.3%)	-
Clinical measurement (25.4%)	-

## Results

### Patient education received on HH

Of the participating patients (*N*=174), 15 (8.6%) reported that they had received patient education on HH, whereas 159 (91.4%) had not. Some of those answering ‘yes’ also described the content in their own words. The content of the received patient education was related to the significance of good HH (frequency=3), the role of the patient in taking care of their own HH independently (frequency=1), handwashing (frequency=2), the use of hand disinfection preparation (frequency=5), the patient's infusion hose (frequency=1), wound care (frequency=1), and ‘everything in general’ (frequency=2). One of the participants (answering ‘yes’) had written:

‘I was reminded about maintaining good hand hygiene when moving around the hospital area. However, the staff members did not adhere to that themselves.’

Three participants who had answered ‘no’ (i.e. they had not received patient education on HH) wrote that they had seen written instructions on the wall. These responses were nevertheless calculated as ‘no’, because it was the perception of the patient that they had not received patient education.

### Extent to which NSCGs were used in accordance with standard precautions

Of the participating 174 patients, 24 reported that one procedure was conducted for them, 22 reported two procedures, 37 reported three procedures, 34 reported four procedures, and 57 reported five procedures. Thus, the data included 600 care, examination or test procedures conducted for the patients.

Table I shows the procedures (*N*=600) conducted for the patients (*N*=174), the need to use NSCGs during each procedure according to the standard precautions for infection prevention and control, and the number of procedures conducted correctly and incorrectly according to the standard precautions. In total, 18 different procedures/procedure types were performed. The use of NSCGs was always correct for six procedures. NSCGs were always used incorrectly when putting a nasal oxygen cannula on the patient; however, there was only one case of this kind. For 13 procedures, the use of NSCGs was correct in >50% of situations.

Table II compiles unnecessary and insufficient use of NSCGs during the procedures conducted for the patients. Unnecessary use of NSCGs was more common (81.3–25.4%) than insufficient use (27.9–12.9%).

### Association between the correct use of NSCGs and the procedure

Only procedures mentioned >15 times were included in this analysis (*N*=515). Chi-squared test showed a significant association between the procedure/procedure type conducted for the patient and the correct use of NSCGs (Table I) [χ^2^ (7)=60.219; *P*<0.001].

### Association between the correct use of NSCGs and the number of HCWs attending the procedure

Cross-tabulation of the correct and incorrect use of NSCGs and whether the procedure (*N*=600) was conducted alone or in the presence of other HCWs is presented in Table III. Chi-squared test showed no significant association between the two variables [χ^2^ (1)=1.111; *P*=0.292].

**Table III:** Cross-tabulation of correct and incorrect use of non-sterile clinical gloves (NSCGs) and whether the procedure was conducted alone or in the presence of other healthcare workers (HCWs) (*N*=600^a^)

Correct use of NSCGs/whether the procedure was conducted alone or in the presence of other HCWs	Incorrect	Correct
In the presence of other HCWs	50 (46.7%)	57 (53.3%)
Alone	183 (41.1%)	262 (58.9%)

a Missing data: frequency=48 (8.0%).

The numbers of HCWs attending the care, examination or test procedures are presented in the supplementary material. In a logistic regression analysis, the number of HCWs did not explain the correct use of NSCGs significantly (odds ratio 0.836, 95% confidence interval 0.672–1.040; *P*=0.108).

## Discussion

### Consideration of the findings

The aim of this study was to explore HCW adherence to patient education regarding HH and the correct use of NSCGs in an observation unit as observed by patients. Only a few patients reported having received patient education regarding HH. A similar result was found by Hammoud *et al.* [5], whose review revealed a low percentage of patient education on infection control measures. Hammoud *et al.* recommended that hospital staff should educate patients on infection control. The present findings were surprising because, according to the standard precaution guidelines of the study hospital, all patients and their visitors must be guided to use an alcohol-based hand rub, and the hygiene instructions for hospital patients must be discussed with the patient.

In expansive responses, some patients mentioned seeing written HH instructions on the wall. However, they had chosen the option ‘no’ when asked if they had received patient education on HH. Although the number of these cases was small, it is important to reflect upon this observation. It may be possible that the HCWs relied too much on the power of the written material, but it is clearly insufficient. To enhance the quality of patient education on infection prevention and control, there is a need to explore the pedagogical principles of effective patient education, and create evidence-based ways to educate patients and visitors. For example, the teach-back method has been shown to have positive effects in a wide range of healthcare outcomes [19,20]. Teach-back can be used when teaching a new skill by asking the patient to demonstrate the new skill so that the HCW can assess the patient's understanding and mastery [21]. It is important to improve the general motivation of HCWs to educate patients and visitors, by explaining and discussing the role and significance of good patient education in infection prevention and control.

On assessment of the extent of correct and incorrect use of NSCGs by HCWs, a significant association was found between the procedure/procedure type conducted for the patient and the correct use of NSCGs. There were 18 different procedures (or procedure types) in the data. Overall, unnecessary use was more common than insufficient use (81.3–25.4% vs 27.9–12.9%, respectively) when procedures mentioned <15 times were excluded from the compilation. In all procedures mentioned >15 times, there was both correct and incorrect use of gloves. The use of NSCGs was always correct for six procedures. However, due to the low number of observations (<15) for each of these procedures, no definite conclusions can be drawn.

NSCGs were used unnecessarily in >80% of cases when transferring the patient/helping with moving or with a moving aid and when serving a meal. However, the frequencies of these procedures were only seven and 16, respectively. Thus, no specific conclusions can be drawn.

During the most frequently conducted procedures (i.e. taking a blood sample; clinical examinations; and administering medication, fluids or blood products), NSCGs were used unnecessarily in >40% of cases. Based on the study data, these are the top three procedures needing intervention to de-implement the unnecessary use of NSCGs. This could include, for example, staff education and increased awareness of the correct way to use protective equipment, which have been shown to be effective ways to reduce the overuse of disposable products [13]. Monitoring the frequency of use could be used to motivate learning [14].

A vacuum technique is used when taking blood samples at the study hospital, as the risk for blood contamination has been found to be minor, whereas blood contamination is possible, for example, when inserting, caring for and removing a cannula. Thus, the instruction is that NSCGs should not be used when taking blood, and this is how HCWs at the study hospital are educated to take blood. It is, however, possible that in spite of this education, some HCWs may feel it necessary to protect themselves when taking blood, even when using the vacuum technique. This needs to be discussed with the HCWs, so they have an opportunity to share their possible concerns and be assured of their safety.

To the authors' knowledge, this is the first study to cover this topic. Studies using external observations [6,8,10] or HCW interviews [9] as data collection methods have also reported on the unnecessary use of NSCGs. The present study increases knowledge about how patient education regarding HH and HCW use of NSCGs appear from the patient perspective.

According to the study data, correct and incorrect use of NSCGs was not associated with whether the procedure was conducted alone or in the presence of other HCWs, and on logistic regression analysis, the number of HCWs did not explain the correct use of NSCGs significantly. This is in contrast to the finding of Monsalve *et al.* [15], who reported that HCW adherence to HH was associated with the number of nearby HCWs. This finding was encouraging, as it showed that HCWs at the study hospital act in the same way (correctly or incorrectly) both when working alone or when working with others.

In this study, almost 60% of NSCG use was unnecessary. In the observation unit, this would mean the consumption of 80,000 unnecessary pairs of NSCGs per year, with unnecessary financial costs. Moreover, the environmental burden [12] is always evident when using disposable gloves.

Further research is needed on the effectiveness of educational interventions supporting the de-implementation of unnecessary use of NSCGs and methods to monitor the use of NSCGs.

### Strengths and limitations

A strength of this study was that patients were offered an opportunity to report on how patient education regarding HH and HCW use of NSCGs appear from the patient perspective. The questionnaire was self-developed and it had not been tested before, which represents a limitation of this study. However, before starting data collection, the research panel, consisting of patients and clients, commented on the information sheet and the questionnaire, and, based on their comments, amendments were made to ensure the relevance and clarity of these documents.

The correctness of NSCG use was determined by the investigators based on the hospital's standard precautions on infection prevention and control, which are based on instructions from the World Health Organization [1]. This enhances the validity of the evaluation.

Another limitation of this study was that the patients were recruited on weekdays alone, and had to be Finnish-speaking. Although the sample does not represent all the patients in the observation unit, the findings produced up-to-date knowledge for use in the development of patient education regarding HH, and in the de-implementation process of any unnecessary use of NSCGs which was one goal of this study. Another goal was to test the method of data collection based on the reports by the patients. The response rate was 87%, which shows that patients were willing and capable of answering the questions in this type of study. The participants were recruited with the help of an assistant head nurse, who indicated patients suitable for the study after careful consideration regarding the condition of each patient. The selected patients were not, for example, in a state of mental confusion, and as they were on an observation ward, an actual state of emergency was not present. The reliability of patient observations was not tested, which represents a further limitation of this study, and it is possible that some of the participants may not have understood everything that was happening. However, only a few simple questions were presented, and it is considered that, despite being laypersons, the participants were capable of answering them sufficiently reliably. The patients were not supposed to observe whether the gloves were used correctly, but only whether or not they were used.

Regarding the generalizability of the findings, similar results may have been found in a similar type of observation unit at any university hospital. However, the study was conducted at a single hospital and included only 174 patients, which are limitations impacting the generalizability of the findings. The study was conducted in an observation unit where the priorities of staff may differ from other clinical areas. The staff of an observation ward often focus on tasks such as measurement and support of vital signs and pharmacological treatments, and they may not always see patient education on HH as being of such importance. This explains why the findings cannot be generalized to other patient areas.

There were missing data because, in some cases, the patient had not indicated the procedure or the answer was unclear. To ensure reliable analysis when exploring the association between the correct use of NSCGs and the procedure, only procedures mentioned >15 times were included in this analysis.

The Strengthening the Reporting of Observational Studies in Epidemiology (STROBE) guidelines [22] were followed to ensure explicit and comprehensive reporting of the study.

In conclusion, patients in the observation unit reported that they rarely received patient education regarding HH. Deviations from the standard precautions on infection prevention and control in the use of NSCGs were found. However, the correct use of NSCGs was associated with procedure/procedure type, although it was not associated with the number of HCWs involved in a procedure. In addition, more procedures occurred with unnecessary use of NGCGs compared with insufficient use. Interventions for HCWs are needed to support routine patient education regarding HH and the evidence-based use of NSCGs.

## CRediT authorship contribution statement

**Hanna-Leena Melender:** Conceptualization, Data curation, Formal analysis, Funding acquisition, Investigation, Methodology, Software, Validation, Visualization, Writing – original draft, Writing – review & editing. **Elina Koota:** Conceptualization, Funding acquisition, Investigation, Methodology, Writing – review & editing. **Katariina Kainulainen:** Conceptualization, Funding acquisition, Methodology, Writing – original draft, Writing – review & editing. **Karoliina Aho:** Conceptualization, Funding acquisition, Methodology, Writing – review & editing. **Marja Mäkinen:** Conceptualization, Funding acquisition, Investigation, Methodology, Project administration, Resources, Writing – review & editing. **Johanna Kaartinen:** Conceptualization, Funding acquisition, Methodology, Project administration, Resources, Supervision, Writing – review & editing.

## Ethical approval

The HUS Regional Committee on Medical Research Ethics was contacted regarding the need for an application for ethical approval. The Committee stated that formal approval was not needed for this study (letter from the HUS Regional Committee on Medical Research Ethics, 20 March 2024). Permission to conduct the study was obtained from the Chief Medical Officer of the hospital (§ 49/2024). Informed consent was obtained from all study participants, and participation was voluntary. The assistant head nurse who indicated patients suitable for study inclusion made careful consideration regarding the condition of each patient before recruitment. Of the recruited patients, many expressed positive feelings about the study and the opportunity to participate. Thus, it is considered that participation did not cause significant harm. All research materials were coded with numbers and not the personal details of the participants. Moreover, assurance regarding participant confidentiality was stated in the information sheet.

## Funding source

This work was funded by state funding for university-level health research (§ 253/2023) (Finland). The funder had no role in the study design; collection, management, analysis and interpretation of data; writing of the report; or the decision to submit the report for publication. Open access was funded by Helsinki University Library.

## Conflict of interest statement

None.
