# Supplementary material for: Patient-reported education regarding hand hygiene and use of non-sterile clinical gloves in an emergency department observation unit
Source: Infect Prev Pract. 2025 Nov 24;8(1):100501. doi: 10.1016/j.infpip.2025.100501 (PMC12861222; doi:10.1016/j.infpip.2025.100501)
Supplement: Multimedia component 1 [file mmc1.docx]

**Appendix A.**

The numbers of HCWs attending the care, examination or test procedures (n = 600*).

| **The numbers**  **of HCWs** | **f** | **%** |
| --- | --- | --- |
| 1 | 453 | 75.5 |
| 2 | 77 | 12.8 |
| 3 | 31 | 5.2 |
| 4 | 19 | 3.2 |
| 5 | 5 | 0.8 |
| 6 | 2 | 0.3 |
| 7 | 1 | 0.2 |

*) Missing data: f = 12 (2.0%)
